# Tétanos localisé sans porte d´entrée apparente: à propos d´un cas à Bamako (Mali)

**DOI:** 10.11604/pamj.2020.36.377.22721

**Published:** 2020-08-31

**Authors:** Hermine Meli, Mikaila Kaboré, Mohamed Aly Cissé, Abdoulaye Zaré, Mariam Soumaré, Yacouba Cissoko, Jean Paul Dembélé, Issa Konaté, Assetou Fofana, Sounkalo Dao

**Affiliations:** 1 Service des Maladies Infectieuses Centre Hospitalier Universitaire du Point G, Bamako, Mali,; 2 Faculté de Médecine et d´Odontostomatologie, Bamako, Mali,; 3 Centre de Recherche et de Formation sur la Tuberculose et le VIH (SEREFO), Bamako, Mali

**Keywords:** Tétanos localisé, Centre Hospitalier Universitaire Bamako, Afrique subsaharienne, Localised tetanus, Bamako University Hospital, sub-Saharan Africa

## Abstract

Le tétanos demeure un problème majeur de santé publique en Afrique subsaharienne. La forme localisée semble rare, contrairement à la forme généralisée suffisamment décrite. Nous rapportons un cas de tétanos localisé sans porte d´entrée apparente, pris en charge dans un service des Maladies Infectieuses à Bamako. Il s´est agi d´une infirmière à la retraite de 59 ans qui n´avait pas fait de rappel de vaccination antitétanique depuis 10 ans, correspondant à la période de son dernier accouchement. Elle avait été référée dans un tableau clinique associant une dysphagie et impossibilité d´ouvrir la cavité buccale. L´histoire de la maladie avait révélé un long itinéraire thérapeutique marqué par de nombreuses consultations spécialisées sans amélioration aucune. Le diagnostic de tétanos localisé sans porte d´entrée apparente avait été retenu après avoir éliminé toute autre affection locale. L´évolution était déjà favorable dix jours après une prise en charge adéquate. Sous diagnostiqué ou méconnu du personnel de santé, le tétanos localisé peut mimer d´autres affections retardant le diagnostic et la prise en charge. La sensibilisation ciblée de la population doit être renforcée en vue du respect scrupuleux du calendrier des rappels vaccinaux.

## Abstract (en)

Tetanus is a major public health problem in sub-Saharan Africa. Localised tetanus is rare, unlike generalized tetanus which has been sufficiently described in the literature. We report a case of localised tetanus with no obvious entry site managed in the Department of Infectious Diseases in Bamako. The study involved a retired nurse aged 59 years who had not undergone tetanus booster immunisation within the last 10 years, corresponding to the date of her last delivery. She was referred to our Hospital with dysphagia associated with inability to open the buccal cavity. Patient’s history was characterized by long-term therapy associated with many specialized consultations without any improvement. The diagnosis of localised tetanus with no obvious entry site was retained after having excluded any other local disorder. Outcome was favorable ten days after adequate management. Underdiagnosed or unknown to health-care providers, localised tetanus may mimic other diseases delaying diagnosis and management. Targeted campaign to build awareness should be implemented in order to improve adherence with immunization schedules.

## Introduction

Le tétanos est une maladie infectieuse non immunisante, due à *Clostridium tetani* et évitable par la vaccination. Il demeure un problème de santé publique dans les pays en développement où il est responsable d´une forte mortalité (30-90%). Le tétanos chez l´adulte peut se présenter sous trois formes: généralisée, localisée à la région anatomique proche de la plaie ou céphalique. La forme localisée est rare en Afrique encore plus, celle sans porte d´entrée apparente. La tétanospasmine, principale toxine produite par la bactérie est responsable de la symptomatologie. Au Mali, le tétanos avec porte d´entrée a été décrite dans 87% [1]. La forme généralisée est de loin la plus fréquente et peut se présenter sous une forme d´emblée ou secondairement généralisée [1]. La forme localisée correspond à une atteinte limitée au site d´inoculation à l´origine d´une contracture ou d´une hypotonie qui peuvent être modérées et persistantes. Elles témoignent d´une immunité partielle vis à vis de la tétanospasmine. Bien que rare, le tétanos localisé constitue un prodrome qui va évoluer vers une forme généralisée [2]. La prophylaxie antitétanique est une stratégie majeure de prévention du tétanos dans de nombreux pays d´Afrique subsaharienne. Au Mali, malgré l´existence d´une politique nationale, la couverture vaccinale du tétanos reste faible [3,4].

## Patient et observation

Nous rapportons un cas de tétanos localisé sans porte d´entrée évidente pris en charge au service des Maladies infectieuses du CHU du point G à Bamako. L´objectif était de décrire ce cas de tétanos localisé qui est une situation peu fréquente en Afrique, paucisymptomatique, à l´origine des erreurs, des retards de diagnostic et de traitements inappropriés. Patiente de 59 ans, infirmière à la retraite qui n´avait pas fait de rappel vaccinal depuis son dernier accouchement, 10 ans auparavant. Elle avait été admise au service des Maladies Infectieuses du CHU du Point G dans un tableau clinique qui associait une dysphagie partielle aux solides et une difficulté d´ouverture de la bouche. L´itinéraire thérapeutique de la patiente révélait quatre (4) consultations dans le service d´odontostomatologie pour atteinte temporo-mandibulaire. Elle y avait reçu de l´Amoxicilline 1 gramme injectable pendant 10 jours, et Tramadol 50 milligrammes pendant 5 jours. Devant la survenue d´épigastralgies, elle avait été adressée à un gastrologue. Une fibroscopie oeso-gastro-duodénale (FOGD) avait été réalisée, puis elle avait été soumise à un traitement symptomatique à base d´oméprazole injectable, 40 mg /jour pendant 10 jours, malgré le résultat de la FOGD sans particularités. Au vu de l´exacerbation de la dysphagie et du trismus, elle sera transportée au service des urgences d´un CHU de la place. L´hypothèse de tétanos localisé y a été posé (diagnostic d´exclusion). Une dose de vaccin antitétanique (VAT), sérum antitétanique (SAT) 1500UI fut administrés et référée au service de Maladies infectieuses du CHU Point G pour plus d´investigations et prise en charge appropriée

A l´admission elle avait un bon état général, la température était 36°C, la pression artérielle 120/80 mmHg, le pouls à 82 pulsations/minute. Le trismus et la dysphagie étaient présents sans paroxysme. On notait par ailleurs une limitation de l´ouverture de la bouche à deux (2) cm et le signe de l´abaisse langue captif présent. Aucune porte d´entrée apparente n´avait été retrouvée. Le diagnostic de tétanos localisé score zéro (0) de Dakar, stade III de Mollaret avait été retenu après avoir éliminé toute autre affection loco-régionale. Sous traitement à base de Diazépam 2 mg/kg/jour en perfusion continue et Métronidazole 30 mg/kg/jr, l´évolution fut marquée 10 jours après par la disparition du trismus et ouverture complète de la bouche. Par ailleurs, le signe de l´abaisse langue captif n´était plus présent.

## Discussion

Depuis la première guerre mondiale, le tétanos localisé est connu et décrit dans les pays industrialisés. Des cas sont rapportés chez des sujets à immunité antitétanique atténuée du fait de leur âge avancé et d´une vaccination ancienne sans rappels [5]. En revanche, en Afrique, le tétanos localisé est peu connu et pourtant c´est dans cette région que l´incidence de cette maladie est plus élevée que le reste du monde. Le tétanos demeure une affection des pays à ressources limitées avec une morbidité et une mortalité élevée au Mali [6]. Paradoxalement dans ces pays, il existe une stratégie de vaccination antitétanique ciblant prioritairement les femmes en âge de procréer, les femmes enceintes, les enfants de moins de 5 ans [3]. Le tétanos localisé se caractérise par des spasmes qui restent localisés à un groupe musculaire proche de la blessure initiale et peuvent durer plusieurs semaines avant de disparaître progressivement. L'extension vers un tétanos généralisé est possible, mais de bon pronostic (moins de 1 % de mortalité) [7]. L´étude de Kakou *et al*. au service de maladies infectieuses du CHU de Treichville en 2004 révèle que le tétanos localisé représentait 1,64 %. L´incidence annuelle a été d´environ deux (2) cas de tétanos localisé contre 123 pour la forme généralisée. L´âge moyen des 2 patients qui présentaient un tétanos dit localisé était respectivement de 22 et 23 ans. Aucun n´avait bénéficié des rappels vaccinaux antitétaniques [8]. Mohamed Amirali *et al*. au Royaume-Uni en 2016 avaient décrit un cas de tétanos chez un homme de 35 ans dont le calendrier de rappel vaccinal n´était pas à jour [9]. Notre patiente était âgée de 59 ans, son dernier rappel vaccinal date de 10 ans correspondant à l´âge de son dernier enfant.

Dans la série de Kakou *et al*., le tétanos céphalique a été observé 11 fois (25%). Il s´est révélé par un trismus, une dysphagie, des cervicalgies, une raideur du cou et des accès de contractures de l´hémiface où siégeait la porte d´entrée. Il y´avait une excellente concordance entre le siège de la porte d´entrée et la localisation du tétanos. En effet, toutes les 11 plaies crânio-faciales ont été à l´origine d´un tétanos céphalique [8]. Notre patiente présentait juste un trismus et une dysphagie. Les cervicalgies et la raideur cervicale étaient absentes. Notons que la bactérie qui est tellurique pénètre dans l´organisme via une effraction cutanée: plaie (accidentelle ou pré existante parfois minime) brûlure, morsure d´animal ou coupure avec un objet contaminé. Le tétanos peut également se transmettre par le biais d´instrument chirurgicaux souillés ou mal stérilisés (pays en voie de développement) [10]. Aucune porte d´entrée n´avait été retrouvée à l´examen physique de cette dernière au cours des consultations d´odontostomatologie, de gastrologie et à l´admission dans notre service. Cette porte d´entrée était probablement minime, et peu visible à l´œil nu. Lorsque *Clostridium tetani* pénètre dans l´organisme au travers d´une effraction cutanée, il produit des exotoxines protéiques neurotropes à l´origine des signes neuro-musculaires [11]. L´absence de porte d´entrée apparente a motivé la patiente à se faire consulter dans un service d´odontostomatologie dès le début du trismus et la dysphagie. N´ayant pas obtenu de suite favorable, elle a été adressée à un gastrologue. La fibroscopie oeso-gastro-duodénale demandée était revenue sans particularité. Aucun diagnostic n´avait été retenu jusque-là, mais un traitement à base d´Oméprazole injectable 40 mg/jour et antalgiques avait été institué. L´exacerbation de la symptomatologie a motivé son orientation dans un service d´Accueil et Urgences où l´hypothèse de tétanos localisé fut posée. Cette forme clinique de tétanos peut mimer d´autres affections [11]. Le retard de diagnostic et/ou de prise en charge adéquat peut aggraver le tableau clinique et entrainer des complications.

## Conclusion

Sous diagnostiqué ou méconnu du personnel soignant, le tétanos localisé est une forme clinique qui existe en Afrique. Il peut se présenter sous un aspect atypique pouvant ainsi retarder le diagnostic et la prise en charge. L´absence d´effraction cutanée ne doit pas faire occulter le diagnostic de tétanos, car toute porte d´entrée n´est pas apparente. Il est urgent de renforcer la sensibilisation ciblée en direction du personnel de santé sur l´existence des formes atypiques du tétanos. A l´endroit de la population sur l´importance du respect des doses de rappels de vaccination antitétanique. L´éviction du tétanos passe incontestablement par la vaccination, le respect du calendrier des doses de rappels, et des soins adéquats post exposition.
